# Microarray-Based Analysis of Methylation Status of CpGs in Placental DNA and Maternal Blood DNA – Potential New Epigenetic Biomarkers for Cell Free Fetal DNA-Based Diagnosis

**DOI:** 10.1371/journal.pone.0128918

**Published:** 2015-07-31

**Authors:** Lotte Hatt, Mads M. Aagaard, Jesper Graakjaer, Cathrine Bach, Steffen Sommer, Inge E. Agerholm, Steen Kølvraa, Anders Bojesen

**Affiliations:** 1 Department of Clinical Genetics, Vejle Hospital, 7100, Vejle, Denmark; 2 Department of Gynecology and Obstetrics, Horsens Hospital, 8700, Horsens, Denmark; 3 Institute of Regional Health research, University of Southern Denmark, 5000, Odense, Denmark; Hospital Authority, CHINA

## Abstract

Epigenetic markers for cell free fetal DNA in the maternal blood circulation are highly interesting in the field of non-invasive prenatal testing since such markers will offer a possibility to quantify the amount of fetal DNA derived from different chromosomes in a maternal blood sample. The aim of the present study was to define new fetal specific epigenetic markers present in placental DNA that can be utilized in non-invasive prenatal diagnosis. We have conducted a high-resolution methylation specific beadchip microarray study assessing more than 450.000 CpG sites. We have analyzed the DNA methylation profiles of 10 maternal blood samples and compared them to 12 1^st^ trimesters chorionic samples from normal placentas, identifying a number of CpG sites that are differentially methylated in maternal blood cells compared to chorionic tissue. To strengthen the utility of these differentially methylated CpG sites to be used with methyl-sensitive restriction enzymes (MSRE) in PCR-based NIPD, we furthermore refined the list of selected sites, containing a restriction sites for one of 16 different methylation-sensitive restriction enzymes. We present a list of markers on chromosomes 13, 18 and 21 with a potential for aneuploidy testing as well as a list of markers for regions harboring sub-microscopic deletion- or duplication syndromes.

## Introduction

Prenatal testing by established invasive procedures such as chorionic villus sampling (CVS) or amniocentesis are associated with a risk of spontaneous abortion in a small number of pregnancies (0,5–1%). Therefore, non-invasive risk-free prenatal testing (NIPT) using fetal-derived genetic material in the maternal blood has been a long-term goal.

The presence of fetal genomic material in the form of fetal cells in the maternal blood circulation was already discovered more than a century ago[1]. Since then many attempts have been made aiming at using fetal cells in the maternal blood as a substitute for amniocytes and chorionic biopsies [2–5]. However, fetal cell based NIPT has been hampered by the very low number of fetal cells in the maternal circulation. An alternative to fetal cells emerged in 1997 when Lo and colleagues discovered cell-free fetal DNA (cffDNA) in the maternal blood, in amounts exceeding 10 times what could be found by fetal cells[6]. Since then, the development of cffDNA analysis for NIPT has been extensive[7]. Several techniques have been developed, but especially Next Generation Sequencing (NGS) of cffDNA has demonstrated impressive results [7–9]. However, NIPT by NGS is still a relatively expensive choice for public health clinics and many still struggle with how to combine NGS-based tests with current clinical practice without leading to an unnecessary increase in cost of the prenatal screening[10].

The search for a more cost–effective method than NGS has fuelled the research for other ways to use and analyze cffDNA. In this respect, utilization of epigenetic differences between maternal blood DNA and cffDNA offers an attractive alternative when searching for fetal chromosomal aberrations [11,12]. The development of microarray-based methylation assays now allows genome-wide quantitative interrogation of methylation levels at the resolution of single CpG sites. Several studies have shown that methylation assays are very effective for screening genomes for differentially methylated CpGs that could potentially be used as fetal specific markers[13–15]

When suitable sites have been found in this way, a more simple technique is needed before applying methylation status as a clinical parameter. Two approaches have been used in this context; Methyl-DNA immunoprecipitation(MeDip) or use of methyl-sensitive restriction enzymes (MSRE) followed by PCR[11,12,15]. Proof of concept has been shown for both approaches, but so far only very few sites have been reported as potential diagnostic markers.

To circumvent this and to further strengthen the chance of finding good diagnostic markers, we have conducted a high-resolution methylation specific beadchip microarray study assessing more than 450,000 CpG sites. We have analyzed the DNA methylation profiles of 10 maternal blood samples and compared them to 12 1^st^ trimesters chorionic (CVS) samples from normal placentas, identifying a number of CpG sites that are differentially methylated in maternal blood cells compared to chorionic tissue. To define differentially methylated CpG sites that can be used with methyl-sensitive restriction enzymes (MSRE) in PCR-based NIPD, we refined the list by selecting sites containing a restriction sites for one of 16 different methylation-sensitive restriction enzymes. In this way, we found 958 differentially methylated CpG sites throughout the genome including possible markers on chromosomes 13, 18 and 21 with a potential for aneuploidy testing as well as a list of markers for regions harboring sub-microscopic deletion- or duplication syndromes.

## Materials and Methods

### Clinical samples

All the samples for the microarray study were sampled from 1^st^ trimester pregnant women, who underwent chorionic villus sampling due to increased risk of trisomy 21, estimated by double test and nuchal translucency testing. For the 10 blood samples, we only used samples from 1^st^ trimester pregnant women with a normal fetus, judged by a normal karyotype on CVS.

We further used 12 CVS samples from normal pregnancies also judged by chromosome analysis. The blood samples and CVS samples were not from the same pregnancy. However, samples were matched on gestational age and gender of the fetus.

The project was approved by the regional committee on health research ethics; The Regional Scientific Ethical Committees for Southern Denmark (Project no: S-20120042). The material used was excess DNA from routine investigation, stored at the biobank at the clinical genetic department at Vejle Hospital. The samples were anonymized and de-identified prior to analysis. The institutional board at the Department of Clinical Genetics and The Regional Scientific Ethical Committees for Southern Denmark therefore waived the need for written informed consent for this study.

### DNA extraction and quantification

#### Blood samples

DNA from blood samples were extracted using a standard salt extraction method. In brief: 40 ml lysis buffer was added to 10 ml of blood collected in EDTA-tubes and centrifuged (30 min., 10.000 RPM, 4°C). The pellet containing leukocytes was collected and washed in 10 ml 0,9% NaCl followed by centrifugation (15 min., 10,000 RPM, 4°C). 3 ml lysis buffer, 250 ul 10% SDS and 25 ul proteinase K (20 mg/ml) was added followed by overnight incubation at room temperature. 1 ml 6 M NaCl was added followed by centrifugation (60 min., 10,000 RPM, 4°C). Isopropanol was finally added and DNA collected in a tube containing TE-buffer.

#### CVS samples

DNA from CVS samples was extracted using a QIAamp DNA Mini kit from Qiagen (QIAGEN Inc., Valencia, CA, USA) according to standard protocol provided by Qiagen.

#### DNA Quantification

DNA samples were quantified with a Nanodrop ND-1000 (Nanodrop Technologies, Wilmington, DE, USA)

### DNA methylation analysis–Infinium microarray analysis

The Illumina Infinium HumanMethylation450 Beadchip Kit (Illumina Inc., San Diego, CA, USA) was utilized for generation of methylation data for all samples. The analysis was done according to standard protocol provided by Illumina. Bisulfit conversion was achieved using a Zymo Research EZ DNA methylation kit (Zymo Research, Irvine, CA, USA). Beadchips were scanned with an Illumina HiScanSQ scanner using standard settings. Initial quality control, background subtraction and raw data normalization were done using the standard algorithms provided in Illumina Genome studio Methylation module v1.0.

### Bioinformatics

Initially, incomplete measurements (i.e. CpG sites for which no β-values were obtained for one or more samples), were filtered out resulting in 471956 CpG sites with β-values for all methylation profiles (Maternal blood cells (MBC); n = 10, Normal CVS (CVS); n = 12). Differences in methylation status between sample groups were evaluated for each CpG-site using the non-parametric Wilcoxon signed-rank test. The P-values were subsequently adjusted for multiple hypotheses testing using Benjamini-Hochberg correction. The resulting false discovery rates (FDRs) were used in combination with a Δβ-value cut-off between sample group means to define differentially methylated CpGs (DMCs). The full dataset has been deposited in the Gene Expression Omnibus (GEO) database (accession number (GSE66210). All data analysis was conducted in R using built-in packages and functions (R version 3.0.2 “Frisbee sailing”) [16]. Heatmaps were produced using the CRAN package “pheatmap” [17]and clustering of samples and methylation sites in heatmaps was obtained with the “complete” clustering method on euclidian distances. All correlation analyses were performed using the non-parametric Spearman method.

#### Identification of epigenetic biomarkers

Identification of potential epigenetic biomarkers requires consistent and comparable methylation of biological replicates within each sample group, and that different sample groups are markedly different in methylation status. Identification of potential biomarkers across all autosomes was achieved using strict filtering conditions thus 10 out of 10 MBC samples should be hypomethylated (β<0.25) and 10 out of 12 CVS samples hypermethylated (β>0.75), or *vice versa*. All potential biomarkers should further overlap with a restriction site for one out of 16 different methylation-sensitive restriction enzymes (S1 Table). Idiogram representation of potential biomarkers was generated using Idiographica [18].

## Results

**Fig 1:**
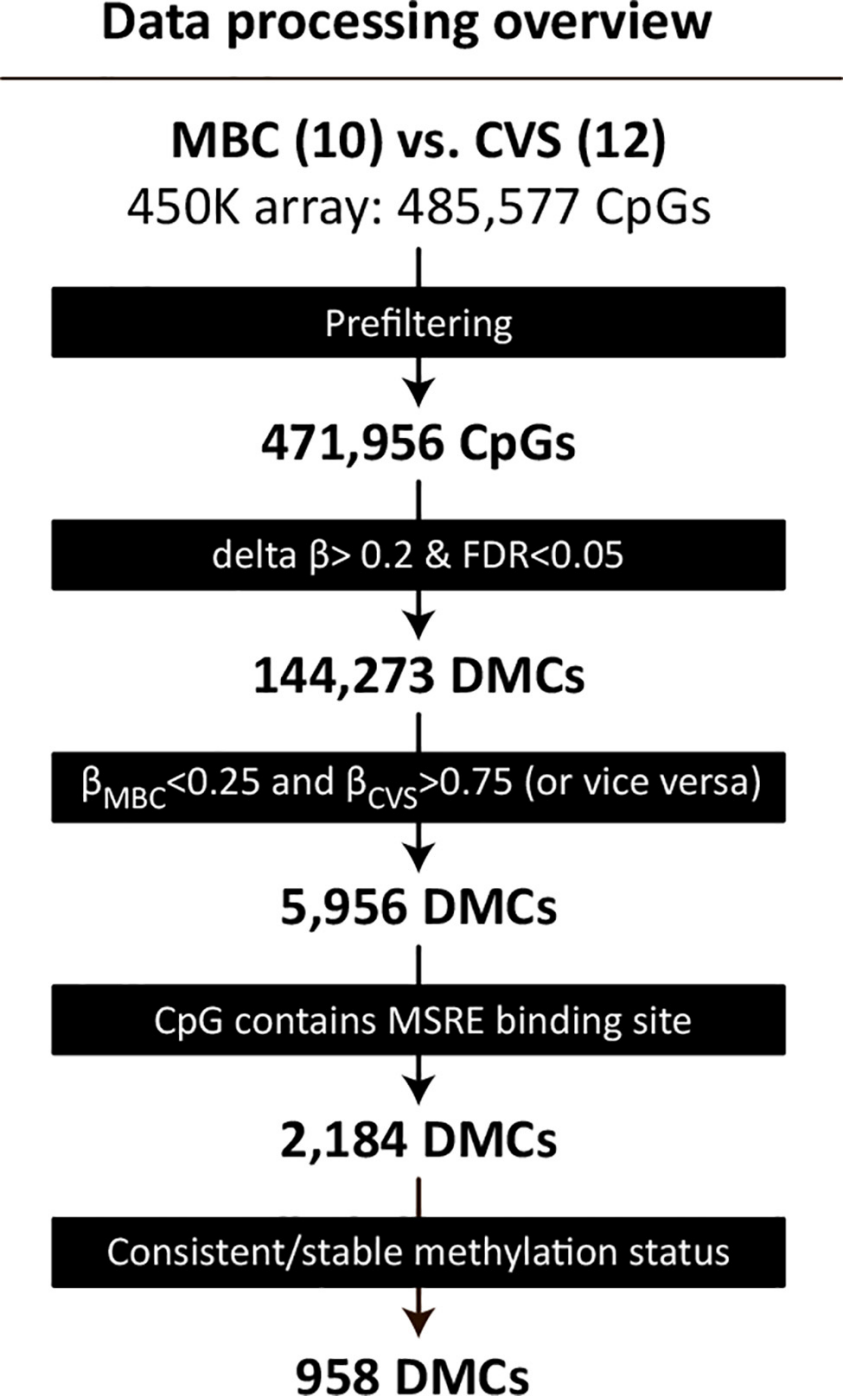
Overview of the Illumina 450K beadchip data filtering process. Methylation data (β-values) for the 485,777 CpGs were filtered in five steps. First, probes for which no β-values were obtained for one or more samples were removed, resulting in a complete dataset for 471,956 CpGs. Second, 144,273 differentially methylated CpGs (DMCs) between maternal Blood Cells (MBC) and Chorionic villus samples (CVS) were identified using a False Discovery Rate (FDR) of <0.05 and delta β>0.2.Third, a more stringent definition of DMCs where MBC samples are hypomethylated (mean β<0.25) and CVS samples are hypermethylated (mean β>0.75), or *vice versa*, dramatically reduced the number of DMCs to 5,956. Fourth, requiring DMCs to overlap a restriction site for a methylation–sensitive restriction enzyme (MSRE), further reduced the number of DMCs to 2,184. Fifth, only DMCs where at least 10 sampleswithin the group pass the hypo- and hyper-methylation thresholds are included, resulting in 958 MSRE-overlapping CpGs with markedly different and highly consistent methylation status between MBC- and CVS-samples.

**Fig 2:**
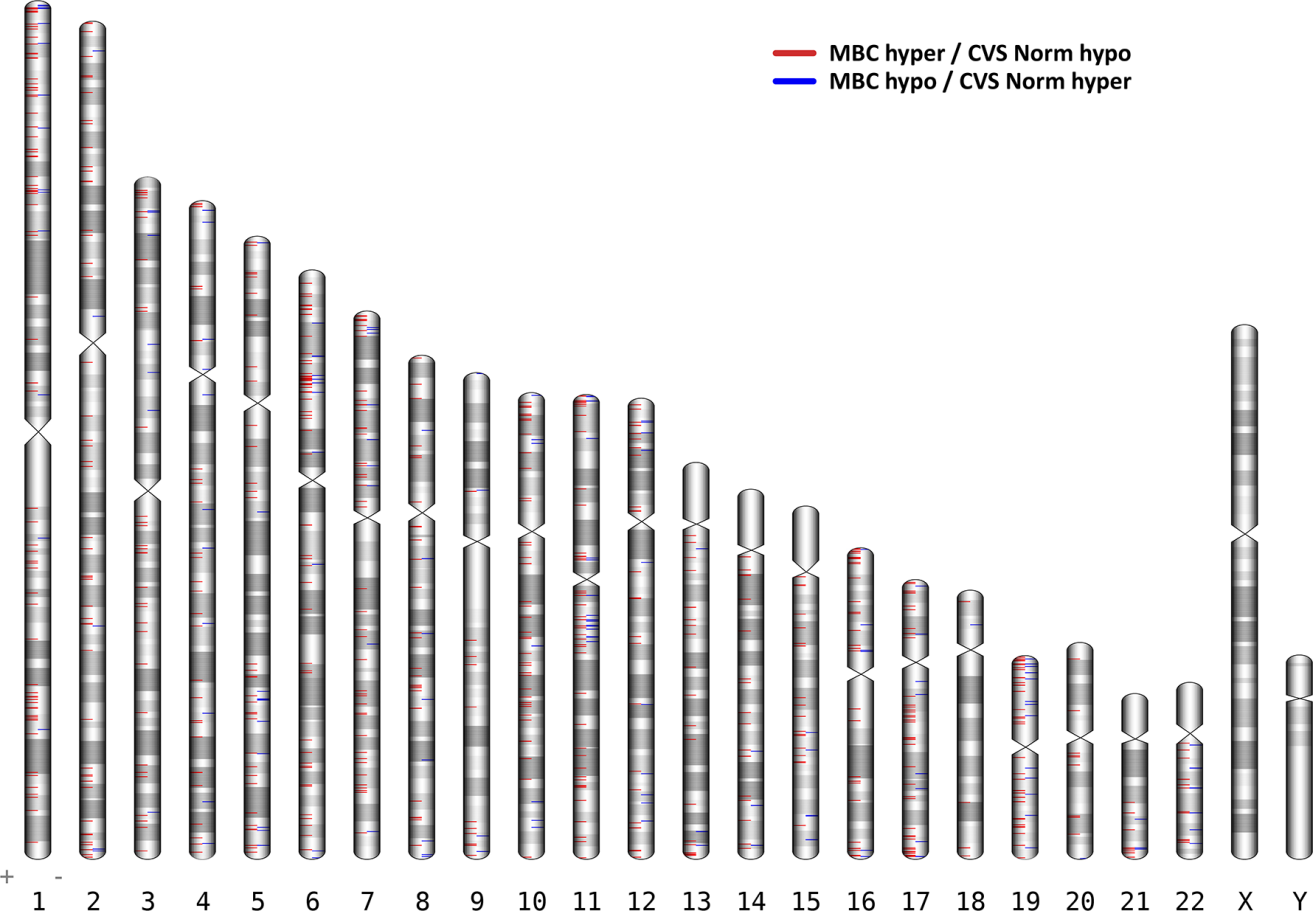
Ideogram of the 958 differentially methylated CpGs (DMCs) between Maternal blood cells (MBC) and Chorionic villus samples (CVS). DMCs that are hypermethylated in MBCs and hypomethylated in CVS are shown in red., whereas DMCs hypomethylated in MBC and hypermethylated in CVS are blue. Chromosomes 13, 18 and 21 have been marked with a black triangle. DMCs on these chromosomes holds the possible use for diagnosis of the three main trisomies; Trisomy 13, 18 and 21.

Initially, we identified 144,273 DMCs (FDR<0.05 and delta β > 0.2) between MBC and CVS samples (Fig 1). However, if DMCs are to be used as fetal specific markers and therefore as diagnostic markers for fetal aneuploidies, the difference in methylation needs to be profound, ideally unmethylated contra fully methylated. Thus, we defined a more strict threshold for hypomethylation (β<0.25) or hypermethylation (β>0.75) thereby reducing the number of DMCs to 5,956 (S2 Table). In order to define sites more suitable for NIPT by use of restriction endonucleases, we furthermore filtered the 5,956 DMCs to only include sites that contain a restriction site for one out of 16 different methylation-sensitive restriction enzymes (MSRE). Finally, strong biomarkers should be consistently methylated across most if not all biological samples within each sample group (MBC or CVS). Consequently, only DMCs where at least 10 of out 12 samples pass the hypo- or hyper-methylation thresholds are considered plausible biomarkers, resulting in 958 DMCs (S3 Table). We found these DMCs to be dispersed throughout the entire genome with possible epigenetic biomarkers on all chromosomes as can be viewed in the ideogram in Fig 2.

**Fig 3:**
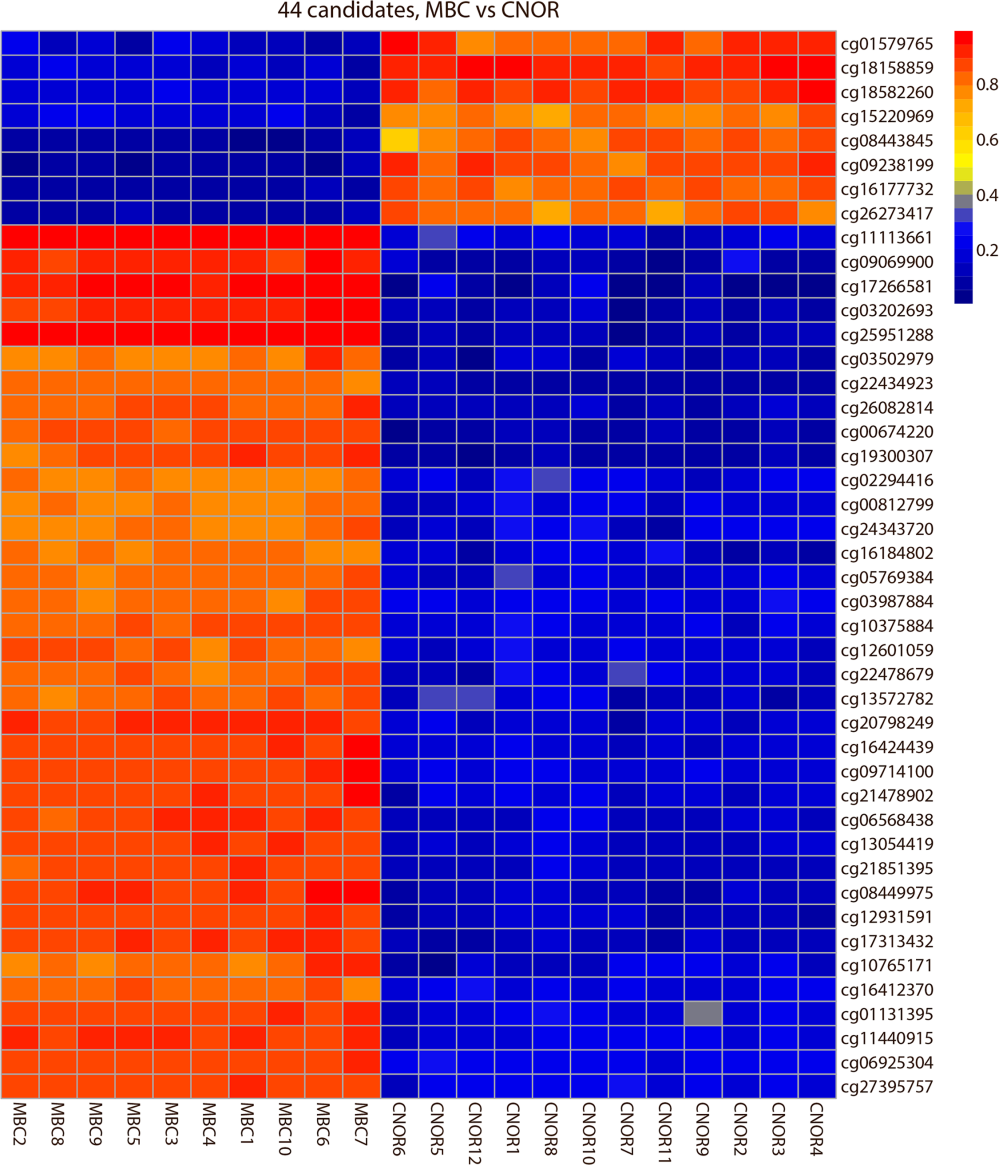
Heatmap visualization of the DNA methylation of 44 differentially methylated CpGs (DMCs) on Chromosome 13, 18 and 21. Rows represent CpG targets, columns represent samples. Each cell is colored according to the level of methylation (β-value).Blue; low β-values, yellow; intermediate β-values, red; high β-values.

Searching for markers that could be used for a possible diagnosis of the common aneuploidies trisomy 21, trisomy 18 and trisomy 13 initially, we found 12 DMCs on Chromosome 21, 6 DMCs on Chromosome 18 and 26 DMCs on Chromosome 13 as listed in Table 1. The consistency of these 44 DMCs can be seen in the heatmap in Fig 3. For all three chromosomes we found DMCs that where hypermethylated in the MBC samples and hypomethylated in the CVS samples as well as vice versa, where the DMCs were hypomethylated in MBC samples and hypermethylated in CVS samples. However, generally we found more sites that were hypermethylated in the MBCs samples and hypomethylated in the CVS samples as can also be visualized in the ideogram presenting the DMCs in all chromosomes (Fig 2).

**Table 1:** Identified candidate biomarkers on chromosome 13, 18 and 21. The table lists for each CpG target the CpG target identification number (illumina), chromosome, nearest gene name, mean methylation levels(β-values) for Maternal Blood Cells(MBC) and Chorionic Villus Samples(CVS), the difference (delta β) between MBC and CVS, and the restriction site for a methylation-sensitive restriction enzyme(MSRE).

CpG Target ID	Chromosome	Gene name	CVS mean	MBC mean	diff. MBC CVS	refseq	MSRE
**cg00674220**	**13**	**USP12**	**0.09**	**0.86**	**0.77**	**NM_182488**	**AciI**
**cg00812799**	**13**	**IL17D**	**0.19**	**0.79**	**0.59**	**NM_138284**	**HhaI**
**cg01131395**	**13**	**LRCH1**	**0.21**	**0.88**	**0.66**	**NM_001164211**	**HpyCH4IV**
**cg02294416**	**13**	**DIAPH3-AS1**	**0.21**	**0.79**	**0.58**	**NR_046539**	**HpyCH4IV**
**cg03202693**	**13**	**MCF2L**	**0.11**	**0.92**	**0.82**	**NM_001112732**	**HpaII**
**cg03502979**	**13**	**ADPRHL1**	**0.11**	**0.80**	**0.68**	**NM_138430**	**HpaII**
**cg05769384**	**13**	**GTF2F2**	**0.19**	**0.82**	**0.63**	**NM_004128**	**HpyCH4IV**
**cg06568438**	**13**	**NDFIP2-AS1**	**0.15**	**0.89**	**0.73**	**NR_046685**	**AatII**
**cg09069900**	**13**	**F10**	**0.11**	**0.91**	**0.80**	**NM_000504**	**AciI**
**cg09238199**	**13**	**COL4A2-AS1**	**0.86**	**0.06**	**0.79**	**NR_046583**	**HpyCH4IV**
**cg10765171**	**13**	**BASP1P1**	**0.16**	**0.83**	**0.67**	**NR_033774**	**BstUI.1**
**cg11440915**	**13**	**LMO7**	**0.20**	**0.90**	**0.70**	**NM_005358**	**AciI**
**cg12601059**	**13**	**OR7E37P**	**0.18**	**0.83**	**0.65**	**NR_002163**	**HpaII**
**cg13054419**	**13**	**MEDAG**	**0.16**	**0.87**	**0.72**	**NM_032849**	**HpaII**
**cg16184802**	**13**	**PCDH9-AS3**	**0.16**	**0.81**	**0.65**	**NR_046636**	**HpyCH4IV**
**cg17313432**	**13**	**MYO16-AS1**	**0.12**	**0.89**	**0.76**	**NR_047700**	**HpaII**
**cg18158859**	**13**	**EFNB2**	**0.93**	**0.16**	**0.76**	**NM_004093**	**HpaII**
**cg18582260**	**13**	**PARP4**	**0.90**	**0.18**	**0.72**	**NM_006437**	**HhaI**
**cg19300307**	**13**	**STK24**	**0.08**	**0.87**	**0.79**	**NM_001032296**	**HpyCH4IV**
**cg20798249**	**13**	**MLNR**	**0.17**	**0.90**	**0.74**	**NM_001507**	**AciI**
**cg21478902**	**13**	**COL4A1**	**0.17**	**0.87**	**0.70**	**NM_001845**	**BstUI.1**
**cg21851395**	**13**	**MIR5006**	**0.14**	**0.87**	**0.73**	**NR_049803**	**HpyCH4IV**
**cg22478679**	**13**	**ADPRHL1**	**0.18**	**0.83**	**0.65**	**NM_138430**	**HpaII**
**cg26082814**	**13**	**ADPRHL1**	**0.13**	**0.84**	**0.70**	**NM_138430**	**AciI**
**cg26273417**	**13**	**EFNB2**	**0.81**	**0.10**	**0.72**	**NM_004093**	**AciI**
**cg27395757**	**13**	**C13orf35**	**0.21**	**0.87**	**0.66**	**NM_207440**	**AciI**
**cg03987884**	**18**	**HMSD**	**0.21**	**0.82**	**0.61**	**NM_001123366**	**BstUI.1**
**cg12931591**	**18**	**TGIF1**	**0.13**	**0.88**	**0.75**	**NM_003244**	**AciI**
**cg13572782**	**18**	**MBP**	**0.17**	**0.82**	**0.65**	**NM_001025081**	**HpaII**
**cg16177732**	**18**	**VAPA**	**0.84**	**0.08**	**0.75**	**NM_003574**	**AciI**
**cg17266581**	**18**	**MBP**	**0.08**	**0.95**	**0.86**	**NM_001025081**	**HpaII**
**cg25951288**	**18**	**NFATC1**	**0.10**	**0.97**	**0.87**	**NM_006162**	**HpaII**
**cg01579765**	**21**	**HSF2BP**	**0.87**	**0.15**	**0.72**	**NM_007031**	**ClaI**
**cg06925304**	**21**	**COL6A2**	**0.23**	**0.87**	**0.65**	**NM_001849**	**HpaII**
**cg08443845**	**21**	**RUNX1**	**0.82**	**0.07**	**0.74**	**NM_001001890**	**AciI**
**cg08449975**	**21**	**DSCR8**	**0.12**	**0.90**	**0.77**	**NM_032589**	**BstUI.2**
**cg09714100**	**21**	**SIK1**	**0.19**	**0.89**	**0.69**	**NM_173354**	**HpaII**
**cg10375884**	**21**	**IFNAR2**	**0.19**	**0.84**	**0.66**	**NM_000874**	**HpaII**
**cg11113661**	**21**	**ICOSLG**	**0.19**	**0.97**	**0.78**	**NM_015259**	**EagI.1**
**cg15220969**	**21**	**SIK1**	**0.79**	**0.18**	**0.62**	**NM_173354**	**HpaII**
**cg16412370**	**21**	**HSF2BP**	**0.21**	**0.83**	**0.62**	**NM_007031**	**BstUI.1**
**cg16424439**	**21**	**ICOSLG**	**0.17**	**0.89**	**0.72**	**NM_015259**	**HpaII**
**cg22434923**	**21**	**PTTG1IP**	**0.08**	**0.81**	**0.73**	**NM_004339**	**AgeI**
**cg24343720**	**21**	**CLDN17**	**0.19**	**0.78**	**0.59**	**NM_012131**	**HpyCH4IV**

Apart from trisomy 13, 18 and 21, the DMCs are also located in other genetic regions of interest. The list of markers includes sites in which deletions causes known syndromes (e.g. cri-du-chat, Smith-Magenis, Prader-willi/Angelman). We here found a number of potentially interesting DMC’s which are listed in Table 2.

**Table 2:** Identified epigenetic biomarkers located in micro-deletion-syndromes. Differentially methylated CpG sites(DMCs))between Maternal Blood Cells(MBCs) and Chorionic Villi samples(CVS) located in DNA regions underlying micro-deletion-syndromes. The table lists chromosome, start, end and region size for the deletion syndromes, and numbers of identified DMCs in each region.

Chromosome	Start	End	Region size	Disease	Number of DMCs containing MSRE in region
**chr4**	0	4500000	4500000	Wolf-Hirschhorn syndrome associated region	7
**chr5**	9800000	33800000	24000000	Cri-du-Chat syndrome associated region	6
**chr11**	0	2800000	2800000	Beckwith-Wiedemann syndrome associated region	12
**chr12**	0	35800000	35800000	Pallister-Killian syndrome associated region	17
**chr15**	8700000	33600000	24900000	Prader Willi syndrome associated region	4
**chr15**	8700000	33600000	24900000	Angelman syndrome associated region	4
**chr17**	0	3300000	3300000	Miller-Dieker lissencephaly syndrome associated region	4
**chr17**	16000000	22200000	6200000	Smith-Magenis /dup(17)(p11.2p11.2) syndrome associated region	1
**chr17**	31800000	38100000	6300000	Renal cysts and diabetes (RCAD) associated region	5
**chr22**	14700000	17900000	3200000	Cat-eye syndrome associated region	1
**chr22**	17900000	25900000	8000000	dup(22)(q11.2q11.2) syndrome associated region	5
**chr22**	17900000	25900000	8000000	DiGeorge syndrome 1/ Velocardiofacial syndrome associated region	5

### Data quality

**Fig 4:**
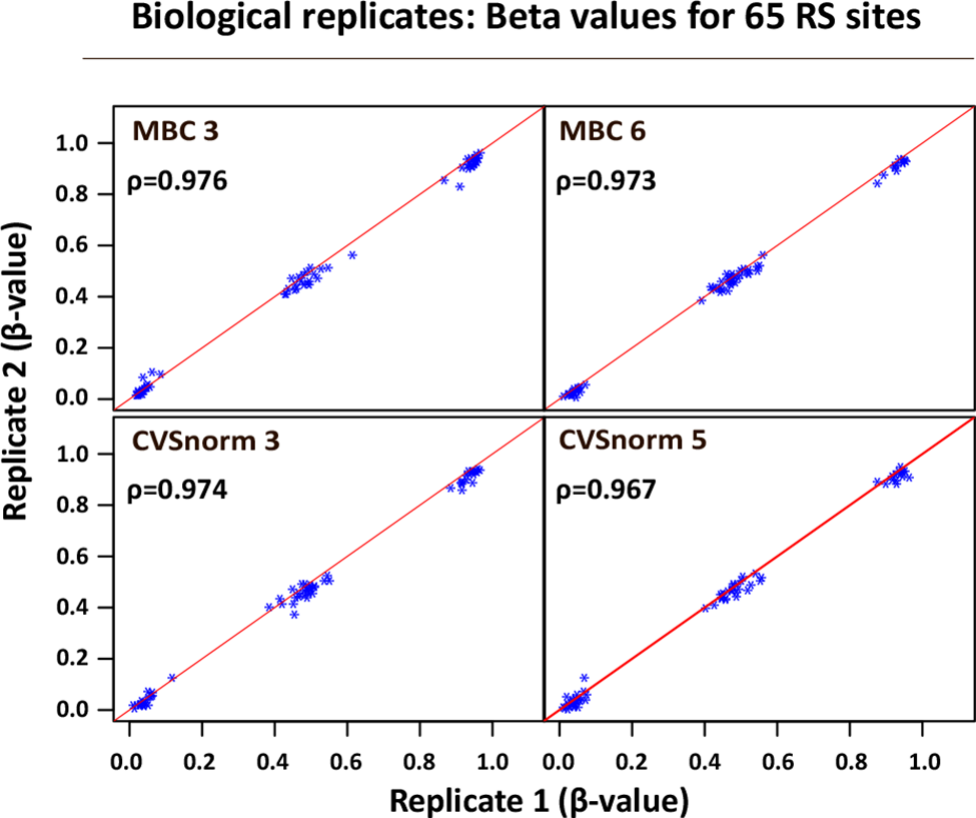
Scatterplot displaying data reproducibility. The beadchip encompass 65 highly polymorphic single nucleotide polymorphisms (SNPs) of the β-vaules for 65 polymorphic SNPs for replicate samples. 4 samples were analysed in duplicates. Two MBC- and two CVS-samples underwent independent bisulphite conversion and were analysed on different bead chip arrays. Spearman correlation coefficient (ρ) for each pair of replicates is shown. Diagonal red line represents complete correlation (ρ = 1.00). Methylation levels (β-value) of 0,0, 0.5, and 1.0 shows clear distinct patterns of homozygous or heterozygous methylation.

We validated the reproducibility of the array analyzes using technical replicates, 2 for each of the sample groups: MBC and CVS. Each of these 4 samples were analyzed in duplicates on different bead chip arrays. The microarray beadchip encompass 65 probes for highly polymorphic single nucleotide polymorphisms (SNPs). When we compared the measured methylation status, (which for the 65 polymorphic probes correspond to genotype) between replicates, we find that the methylation status for each of these 65 sites is highly comparable between replicates (Spearman rho for each pair of replicates; 0.96 to 0.98) for all samples (Fig 4). These data demonstrate a high degree of validity and reproducibility in our array analyses.

We further compared our data to the DMCs found by Bunce *et al*. 2012. They also applied the illumina beadchip but in a smaller scale, assessing only 27.000 CpG sites. In this study they filtered the data only assessing chromosome 13, 18, 21, X and Y and found 47 differentially methylated CpGs. We could in our own data set obtain values for 44 of these 47 CpG sites and we found that all 44 sites where likewise differentially methylated in our data set (p<2.074e-06) (Heatmap presented in S1 Fig).

## Discussion

The primary aim of our study was to identify CpG sites with different methylation in maternal blood leukocytes compared to fetal derived placental DNA, that might ultimately be used as cffDNA specific markers in non-invasive prenatal diagnosis of fetal aneuploidies. Especially CpG-sites where the maternal DNA fraction is hypomethylated and the fetal/placental DNA fraction is hypermethylated are of interest, since these sites—if they overlap a recognition site for a MSRE—enables digestion of the maternal DNA whereas the fetal DNA is protected from digestion by the methylation group. With subsequent PCR analysis comparing differentially methylated DNA sequences on chromosome 21 compared to a control chromosome, it could be possible to predict if a fetus is diploid or triploid for chromosome 21. Proof of principle of this concept has been shown by Tong et al. in 2010 by an epigenetic chromosome dosage analysis using MSRE and digital PCR. They used the markers HLCS on chromosome 21 and compared it to RASSF1A on Chromosome 3 and ZFX or ZFY on the sex chromosomes. They were able to demonstrate fetal specific chromosome dosage from HLCS and ZFY in this way [11]. Further, Lim et al. were able to detect fetal trisomy 21 by measuring unmethylated U-PDE9A in a nested case control study[19].Women with Trisomy 21 fetuses did have a significant elevated U-PDE9A level compared to controls, but the sensitivity of the U-PDE9A for DS detection was only 77.8% and they had a false positive rate of 5%.

In the present communication we aimed to define more sites suitable for this type of prenatal diagnosis in order to strengthen sensitivity and specificity by increasing the number of investigated sites. By applying strict beta-value thresholds for hyper and hypo-methylation status we have optimized the likelihood that candidate CpG sites are indeed fully methylated or completely unmethylated in the cell free plasma DNA.

To further strengthen the potential use of these CpG sites in NIPD, we have filtered the sites to include only differentially methylated CpG sites encompassing a restriction site for a MSRE. We thereby have the possibility to digest and degrade the fraction of either the maternal or the fetal DNA depending on the methylation status of the two. It is in addition, possible to apply the DMCs without an MSRE site with other techniques such as methylation specific quantitative PCR. This technique holds the ability to analyze both fetal methylated and unmethylated DMCs[19].

The sites defined in this study therefore hold the possibility to be used as cffDNA markers, although further investigation is needed to validate if these markers can be used as fetal diagnostic markers. We are, however, aware that caution should be taken in the interpretation of microarray based data from CVS samples, since the chorionic villi is a heterogeneous mixture of syncytiotrophoblastic-, cytotrophoblastic-, mesodermal- and fetal endothelial/vascular cells, and the proportion of the different cell populations in the biopsy could be a confounding factor. In the present study, however, we have, within each group analyzed samples from 12 different individuals. We thereby minimize the risk of variability, giving a more representative biological measure. To further limit the risk of confounding factors such as sex and gestational age we have used gestational–age-matched blood samples with an equal distribution of samples with male and female fetuses.

WBC DNA from pregnant women was selected as a proxy for maternal cfDNA in this methylation study. This implies that these sample contain minute amounts of cffDNA that will contribute to the overall methylation data. However, the fraction of cffDNA to maternal genomic DNA should be so small (<1%) that it should not interfere with our chosen DMCs since our threshold value for a hypomethylated CpG is β <0.25 (equals < 25% methylation). In addition, we chose WBC from pregnant women over non-pregnant women, since we speculated that there is possibility that the maternal WBC could have small methylation differences due to the pregnancy and the presence of the fetal allograft.

Many of the previous studies have been focusing on finding markers only on chromosome 13, 18 and 21. However, if epigenetic markers are to be used for NIPT, all chromosomes should be covered. In the diagnosis of trisomic chromosomes or microduplication or microdeletion the use of good markers on control chromosomes are just as important for the correct quantification and diagnosis. Therefore we have chosen to find markers covering the whole genome. The DMCs that we found in the regions of other chromosomal aberrant disorders opens for the possibility that epigenetic markers can be extended to more than trisomic testing. In a recent study from 2014 Xiang *et al*. They found DMCs in the DNA regions underlying Crit-du-chat syndrome and velo-cardio-facial syndrome (VCFS)[20].

In conclusion we present here a comprehensive analysis of the methylation status of CpG sites in maternal blood cells and normal CVS samples. With a strict discrimination of hyper- and hypo-methylated sites, we present a list of markers on chromosomes 21, 18 and 13 and other autosomes. We have furthermore found potential markers for a range of genetic disorders which could potentially be used in future noninvasive prenatal diagnosis. These candidate markers contain one of 16 different MSREs, thereby enhancing the possibility to choose and combine more markers for a more optimized and specific non-invasive prenatal testing in future studies.

## Supporting Information

S1 Fig Heatmap of 44 DMCs found by Bunce et al. We compared our data to the differentially methylated sites (DMCs) found by Bunce et al. 2012. This group also applied the illumina beadchip but in a smaller scale, assessing only 27.000 CpG sites and only assessing chromosome 13, 18, 21, X and Y. They identified 47 differentially methylated CpGs. We could in our own data set obtain values for 44 of these 47 CpG sites and we found that all 44 sites where likewise differentially methylated in our data set (p<2.074e-06). The heat map presents the methylation level of the 44 DMCs from our dataset.
(EPS)

S2 Fig Boxplot of the beta value variability for the 958 DMCs for each of the 4 technical replicates. (EPS)

S1 Table Methylation-sensitive restriction enzymes. (DOC)

S2 Table Differentially methylated CpGs. DMCs were obtained by using strict filtering conditions thus 10 out of 10 MBC samples should be hypomethylated (β<0.25) and 10 out of 12 CVS samples hypermethylated (β>0.75), or *vice versa*. The table lists Illuminas target ID, chromosome, Methylation level as mean β-value for maternal blood cells (MBC), mean β-value for Chorionic samples (CVS), and gene name.
(XLSX)

S3 Table Differentially methylated CpGs containing a site for a methylation sensitive restriction enzyme (MSRE). DMCs were obtained by using strict filtering conditions thus 10 out of 10 MBC samples should be hypomethylated (β<0.25) and 10 out of 12 CVS samples hypermethylated (β>0.75), or *vice versa*. The table lists Illumina target ID, chromosome, Methylation level as mean β-value for maternal blood cells (MBC), mean β-value for Chorionic samples (CVS), gene name and site for methylation sensitive restriction enzyme (MSRE)
(XLSX)
