# Supplementary material for: Microarray-Based Analysis of Methylation Status of CpGs in Placental DNA and Maternal Blood DNA – Potential New Epigenetic Biomarkers for Cell Free Fetal DNA-Based Diagnosis
Source: PLoS One. 2015 Jul 31;10(7):e0128918. doi: 10.1371/journal.pone.0128918 (PMC4521692; doi:10.1371/journal.pone.0128918)
Supplement: S1 Table — (DOC) [file pone.0128918.s003.doc]

|  | Methylation-Sensitive  Restriction Enzymes | Restriction site |
| --- | --- | --- |
| 1 | AatII | GACGT/C |
| 2 | AciI | CCGC(-3/-1) |
| 3 | AfeI | AGC/GCT |
| 4 | AgeI | A/CCGGT |
| 5 | BstUI | CG/CG |
| 6 | ClaI | AT/CGAT |
| 7 | EagI-HF™ | C/GGCCG |
| 8 | HhaI | GCG/C |
| 9 | HpaII | C/CGG |
| 10 | HpyCH4IV | A/CGT |
| 11 | KasI | G/GCGCC |
| 12 | PaeR7I | C/TCGAG |
| 13 | PmlI | CAC/GTG |
| 14 | SacII | CCGC/GG |
| 15 | SalI-HF™ | G/TCGAC |
| 16 | SnaBI | TAC/GTA |
